# Retrocaval Ureter With Ureteric Stones: A Case Report

**DOI:** 10.7759/cureus.58826

**Published:** 2024-04-23

**Authors:** Raed Alwadai, Naif A Alqarni, Abdullah Ayed

**Affiliations:** 1 Department of Urology, King Abdullah Hospital, Ministry of Health, Bisha, SAU; 2 Department of Medicine, College of Medicine, University of Bisha, Bisha, SAU; 3 Department of Surgery, College of Medicine, University of Bisha, Bisha, SAU

**Keywords:** hydroureteronephrosis, open surgery, anomalies of inferior vena cava, ureteral stone, retrocaval ureter

## Abstract

The retrocaval ureter is an uncommon anomaly where the ureter passes behind the inferior vena cava. Open surgery had been the gold standard for treatment. We are presenting a case of the retrocaval ureter with ureteral calculi, which was effectively managed by open surgery. A 27-year-old male presented with a nine-month history of flank pain. He had no history of chronic illnesses. Physical examinations and laboratory findings were within normal. A computed tomography (CT) scan was done to confirm the diagnosis of retrocaval ureter with ureteral stones. The subcostal incision was made. Then, the proximal and lower ureter was transected at the point where it went retrocaval. The stones were extracted; then, watertight anastomosis was done. Ultrasound used for the follow-up of the patient for six months showed no hydronephrosis. Retrocaval ureteral may have no symptoms or be linked to nonspecific symptoms. The diagnosis of the retrocaval ureter is frequently delayed. Surgical management is utilized in the majority of cases.

## Introduction

Retrocaval ureter is an uncommon anomaly, in which the ureter deviates medially behind the inferior vena cava, predominantly involving the right ureter [1]. This anomaly was first described by Hochstetter in 1893 [2]. Inferior vena cava anomalies, in general, are rare, occurring in about 8.7% of the population [3]. Retrocaval ureter is a rare anomaly that presents with symptoms most commonly in the third or fourth decade of life with an approximate incidence of 1 in 1000 [4,5]. This condition is more commonly found in males, with the majority of patients presenting with pain in the flank area and hematuria [6]. Open surgery has been the gold standard for the treatment of retrocaval ureter [7]. We are presenting a case of the retrocaval ureter with ureteral calculi, which was effectively managed by open surgery.

## Case presentation

**Figure 1:**
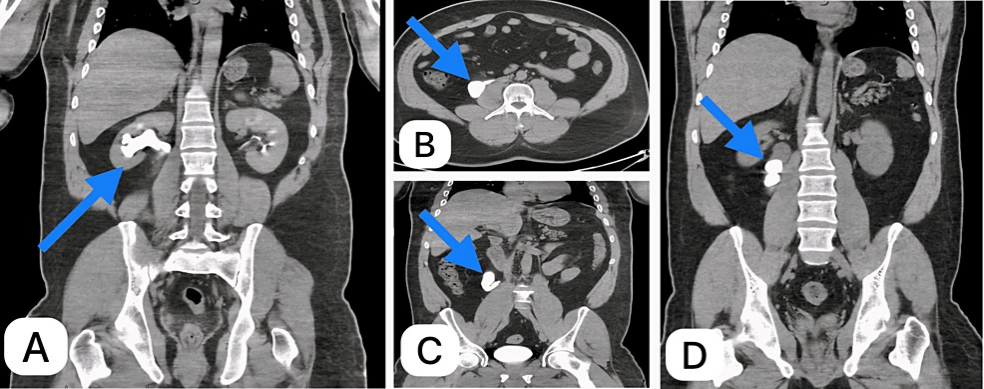
Computed tomography (CT) scan Blue arrows show (A) a coronal image of CT KUB with contrast showing a dilated ureter and renal pelvis, (B) an axial image of CT with contrast delayed film showing a ureter behind the IVC, (C) a coronal image of CT KUB with contrast delayed film showing a dilated upper ureter and hooked-shape ureter, and (D) coronal plain CT KUB showing right upper ureteric stones with a dilated upper ureter KUB, kidney, ureter, and bladder; IVC, inferior vena cava

A 27-year-old Yemeni male presented with a nine-month history of intermittent dull right flank pain. His pain started slowly, was intermittent, did not radiate, got worse over time, and was decreased by muscle relaxants and nonsteroidal anti-inflammatory drugs (NSAIDs). He had no history of chronic illnesses. The general physical examination and focused abdominal examination both showed normal results. The abdomen was soft with no abdominal or renal angle tenderness. The patient appeared to be in good health, with no signs of fever. Additionally, his vital signs were within normal range. The results from the laboratory, such as tests on kidney function and the analysis of urine, came back within normal ranges. Abdominal ultrasound revealed right-sided marked hydroureteronephrosis. A computed tomography (CT) scan with intravenous (IV) contrast confirmed the diagnosis of retrocaval ureter. The CT showed right hydroureteronephrosis with a ureteral calculus located proximal to the retrocaval ureter (Figure 1).

**Figure 2:**
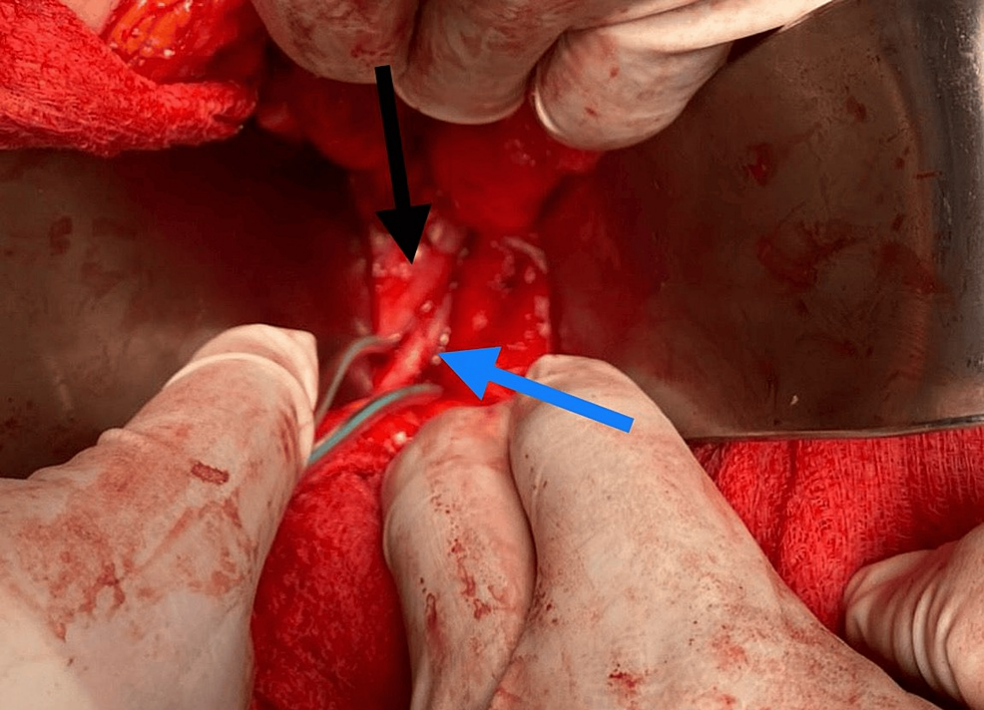
Operative photograph The ureter (blue arrow) coursing behind the inferior vena cava (black arrow)

The patient was admitted to the surgical department, and the patient was counseled for surgery, and full informed consent was obtained. Preoperative workup was within normal ranges. The operation was done under general anesthesia. We performed retrograde pyelography, and we inserted a ureteric catheter. The patient was positioned on his left side at 45° to the left decubitus. A right subcostal incision was made. Colon mobilization was done to expose the ureter. The ureter was traced near the ureteropelvic junction and dissected lower down to the lateral aspect of the inferior vena cava. Then, the proximal ureter was transected at the location where it passed behind the inferior vena cava. The lower end was surgically removed from the back of the inferior vena cava. The ureter ends were positioned in the front of the vena cava. Spatulation of both ends of the ureter was done. The insertion of a double J stent was done; then, watertight anastomosis was done (Figure 2).

**Figure 3:**
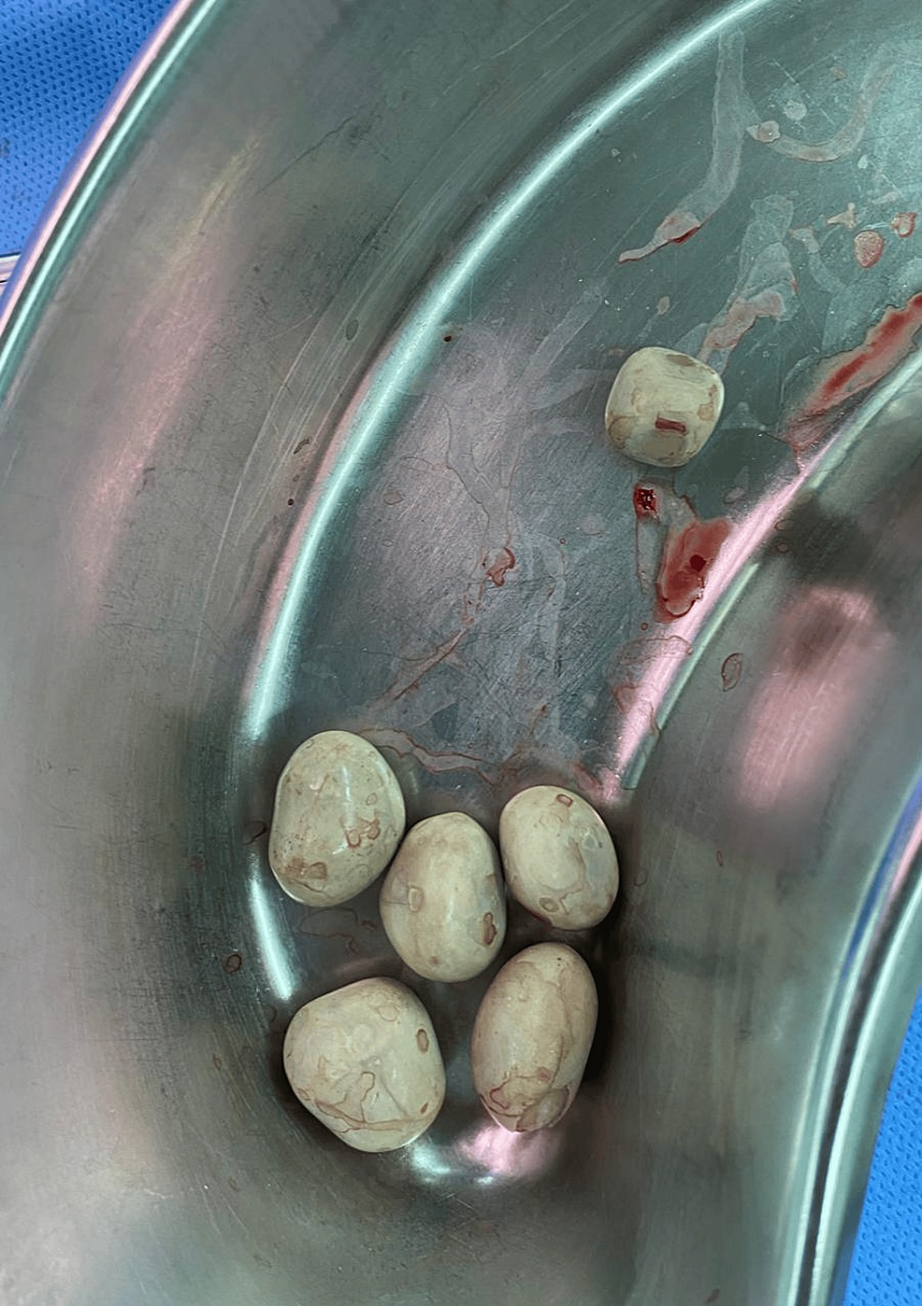
During operation photograph Six extracted stones

The stones were extracted (Figure 3).

Laboratories and abdominal ultrasound were normal. Ultrasound used for the follow-up of the patient for six months showed no hydronephrosis.

## Discussion

Retrocaval ureter is an uncommon genetic abnormality involving the inferior vena cava, where the ureter goes behind it; this occurs due to an irregular development of the infrarenal inferior vena cava from subcardinal veins positioned in front instead of supracardinal veins located behind [8]. It causes the ureter to loop around the inferior vena cava. It is alternatively called the circumaural ureter or ureteral vena cava [9]. This condition has a higher incidence in males than females [10]. Two categories of retrocaval ureter have been identified according to the imaging results. Type 1 includes an inherent abnormality in the growth of the retrocaval section of the ureter leading to a blockage syndrome that needs surgical removal; type 2 is caused by external pressure on the regular ureter in its retrocaval area, which can be treated with plasty instead of removal [1]. One of the primary causes of its delayed diagnosis, and in some cases even after death, is its secretive development [11]. Symptoms typically develop in the third or fourth decade of life as a result of ureteral compression, leading to ureteral kinking and hydronephrosis either from the inferior vena cava pressing against the psoas muscle or from a nonfunctional retrocaval ureteral segment [12]. Symptoms such as flank pain, abdominal pain, and hematuria are determined by the level of ureteral blockage or any associated complications [13]. The obstruction of the ureter can lead to complications such as urinary infection, renal dysfunction, and the formation of stones [14]. The retrocaval ureter is usually diagnosed with a computed tomography (CT) scan, retrograde pyeloureterogram, or intravenous urogram [3]. Possible treatment options for patients include observation for those without symptoms, surgical reconstruction for individuals experiencing hydronephrosis, or nephrectomy of the affected kidney in cases of cortical atrophy [15]. Surgery is essential to maintain kidney function when the urinary tract is completely blocked. Surgical treatment options for the retrocaval ureter include laparoscopic, robotic, or open surgery [16-18]. Although open surgery has been the gold standard for the treatment of retrocaval ureter [7], laparoscopic surgery has been used to treat this condition [19].

## Conclusions

As this is the first case reported from our city, we are working to increase awareness among clinicians. Retrocaval ureter, a condition where the ureter is compressed by the inferior vena cava, may not always exhibit symptoms. However, it can be associated with symptoms such as flank pain, abdominal pain, hematuria, or nonspecific symptoms. Unfortunately, the diagnosis of retrocaval ureter is often delayed. In most cases, surgical treatment options such as laparoscopic, robotic, or open surgery are utilized.
